# Further insights into the phylogeny of facultative parasitic ciliates associated with tetrahymenosis (Ciliophora, Oligohymenophorea) based on multigene data

**DOI:** 10.1002/ece3.10504

**Published:** 2023-09-05

**Authors:** Lihui Liu, Mingyue Jiang, Chunyu Zhou, Bailin Li, Yumeng Song, Xuming Pan

**Affiliations:** ^1^ Key Laboratory of Biodiversity of Aquatic Organisms Harbin Normal University Harbin China

**Keywords:** ciliates, ITS1‐5.8S‐ITS2 rRNA gene, LSU‐rRNA gene, phylogeny, SSU‐rRNA gene, Tetrahymenida

## Abstract

Tetrahymenosis, caused by about 10 *Tetrahymena* species, is an emerging problem inflicting a significant economic loss on the aquaculture industry worldwide. However, in the order Tetrahymenida, there are many unresolved evolutionary relationships among taxa. Here we report 21 new sequences, including SSU‐rRNA, ITS1‐5.8S‐ITS2 rRNA and LSU‐rRNA, genes of 10 facultative parasitic *Tetrahymena* associated with tetrahymenosis, and conduct phylogenetic analyses based on each individual gene and a three‐gene concatenated dataset. The main findings are: (1) All the parasitic and facultative parasitic species cluster in borealis group. (2) With the addition of new sequences, *Tetrahymena* is still divided into three groups, namely the “borealis group”, the “australis group,” and the “*paravorax* group.” (3) the cluster pattern of all the newly sequenced facultative parasitic *Tetrahymena* species shows that members of the “borealis” group may be more susceptible to parasitism. (4) phylogeny based on concatenated genes show that *T. pyriformis*, *T. setosa*, and *T. leucophrys* have close relationship.

## Graphical abstract

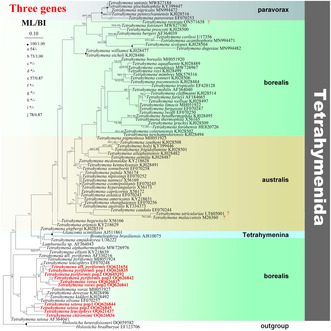

## 1 INTRODUCTION

Ciliates are a highly differentiated group of microbial eukaryotes that inhabit virtually all environments in the world. They play an important role in the microbial food web through transfer of material and energy (Arregui et al., 2002; Azam & Malfatti, 2007; Bai et al., 2020; Caron et al., 2012; Ewa & Sebastian, 2018; Gao et al., 2020; Kathol et al., 2009; Lara et al., 2011; Li et al., 2022; Liu et al., 2020; Luo et al., 2021; Pan et al., 2020; Pilling et al., 2017; Song et al., 2021; Wang et al., 2020; Weisse, 2017; Wu et al., 2021).

*Tetrahymena*, assigned to class Oligohymenophorea, is a pyriform‐shaped ciliate, with buccal cavity equipped with one paroral membrane, and three serially arranged membranelles (Corliss, 1960, 1962, 1965, 1968, 1970, 1971a, 1971b; Lynn, 2008; Pan et al., 2018; Wang et al., 2019). Occasionally, this microorganism can become parasitic and infect a wide range of hosts, including *Channa aurantimaculata*, *Chironomus plumosus*, *Deroceras retioulatum*, *Ictalurus punctatu*s, *Passalus cornutus*, *Poecilia reticulata*, and *Trichia lubomirskii* (Lawhavinit et al., 2002; Lom, 1995; Pan et al., 2022; Prosser et al., 2018; Shenberg, 2003; Simon et al., 2008; Thompson Jr, 1958). Some of them are facultative parasites while others might be obligate parasites (Corliss, 1970; Rataj & Vdacny, 2020). The collective name for diseases affecting many vertebrates and invertebrate hosts caused by *Tetrahymena* is tetrahymenosis. The most pathogenic species of this genus is *T. corlissi*, which often leads to the systemic infection known as “guppy killer disease” (Ferguson et al., 1987; Hatai et al., 2001; Imai et al., 2000).

Traditionally, species identification of *Tetrahymena* is by morphology; however, it has limitations for the non‐expert since it relies on silver staining methods, which require experience and skill (Corliss, 1960). Although most investigations have focused on the identification, pathological features, and infection mechanisms of parasitic *Tetrahymena* (Lawhavinit et al., 2002; Li et al., 2021; Pan et al., 2022), few studies have explored the phylogeny of facultative parasitic or parasitic ciliates. Under‐sampling still continues to cloud the picture for phylogenetic studies based on multi‐gene sequences of facultative parasitic or parasitic *Tetrahymena* (Lawhavinit et al., 2002).

In this work, 21 new sequences of 10 facultative parasitic *Tetrahymena* isolated from fish market in Harbin, China are provided and, for the first time, phylogenies are inferred from individual and concatenated genes (SSU rRNA, LSU rRNA, and ITS1‐5.8S‐ITS2 rRNA). The evolutionary relationships of some facultative parasitic species associated with tetrahymenosis were investigated, in order to provide some theoretical support for the origin and evolution of common parasitic species.

## 2 MATERIALS AND METHODS

### 2.1 Ciliate isolation, observation, and identification

*Tetrahymena* species were collected from live specimens of various fish from fish markets in Harbin, China from June 2021 to Dec 2022. Diagnosis of *Tetrahymena* species were conducted by light microscopical observation under a light microscope (20× and 40× objective) of fresh mount preparations, which revealed the presence of ciliates on the skin, in the surface mucus, in the surface gills and in internal organs, such as the liver. Cells were isolated and monoclonal were established in Petri dishes with the host fish tissues were added as the nutrient source.

Cells were observed and photographed in vivo using bright field and differential interference contrast microscopy (Zeiss, Axio Imager A2). Silver carbonate (Foissner, 1992) and protargol (Wilbert, 1975) staining methods were used to reveal the infraciliature. Protargol was synthesized according to Pan et al. (2013) Counts and measurements of specimens were conducted under magnifications of 100–1250×. Terminology and systematics mainly refer to Gao et al. (2016) and Lynn (2008).

### 2.2 DNA extraction, PCR amplification, and sequencing

Ten cells were separated by micropipette under the stereomicroscope and washed with double distilled water. Cells were then transferred to the Eppendorf tube with small volume of water. They were then starved in nutrient‐free distilled water for 12 h. Total genomic DNA was extracted from cells using Dneasy & Tissue Kit (Shanghai, QIAGEN) according to manufacturer's instructions.

The SSU‐rRNA, ITS1‐5.8S‐ITS2 rRNA and LSU‐rRNA genes were amplified by polymerase chain reaction (see Table 1 for primers). Hi‐fi Taq polymerase (Takara Ex Taq; Takara Biomedicals) to reduce amplification errors.

**TABLE 1:** Primers used in the polymerase chain reactions for ciliate SSU‐rRNA, ITS1‐5.8S‐ITS2 rRNA, and LSU‐rRNA gene in the present study.

Molecular marker	Primer name	Primer sequence (in 5′ to 3′ direction)	References
SSU‐rRNA	Euk‐A	5′‐AAC CTG GTT GAT CCT GCC AGT‐3′	Medlin et al. (1988)
	Euk‐B	5′‐TGA TCC TTC TGC AGG TTC ACC TAC‐3′	Medlin et al. (1988)
LSU‐rRNA	5.8S‐F	5′‐GTA GGT GAA CCT GCG GAA GGA TCA TTA‐3′	Goggin (1994)
	LO‐R	5′‐GCT ATC CTG AGR GAA ACT TCG‐3′	Pawlowski (2000)
ITS1‐5.8S‐ITS2 rRNA	5.8S‐F	5′‐GTA GGT GAA CCT GCG GAA GGA TCA TTA‐3′	Goggin (1994)
	5.8S‐R	5′‐TAC TGA TAT GCT TAA GTT CAG CGG‐3′	Goggin (1994)

PCR condition for amplification of the SSU‐rRNA gene was: denaturation for 5 min at 94°C, followed by 5 cycles of denaturation in the 45 s at 94°C, annealing for 1 min at 45 s at 56°C, extended for 2 min 72°C, and other 25 cycles of denaturation for 45 s at 94°C, annealing 1 min 45 s at 60°C, extended for 2 min at 72°C, and the last one extended for 8 min at 72°C. The ITS1‐5.8S‐ITS2 rRNA gene was amplified as follows: 5 min initial denaturation 94℃, followed by 35 cycles of denaturation for 30s at 94℃, annealing for 45s at 58℃, extension for 1 min at 72℃ and final extension at 72℃ for 10 min. The LSU‐rRNA gene was amplified as follows: 94°C initial denaturation 3 min, followed by 35 cycles of denaturation in the 15 s at 95°C, annealing for 1 min at 55°C, extended for 2 min 72°C, 72°C eventually extends for 10 min. Cloning and sequencing are routine procedures (Zhang et al., 2019). A total of 21 new gene sequences of SSU‐rRNA, ITS1‐5.8S‐ITS2 rRNA, and LSU‐rRNA genes were identified from 10 *Tetrahymena* species.

### 2.3 Data sets and alignment

**FIGURE 1:**
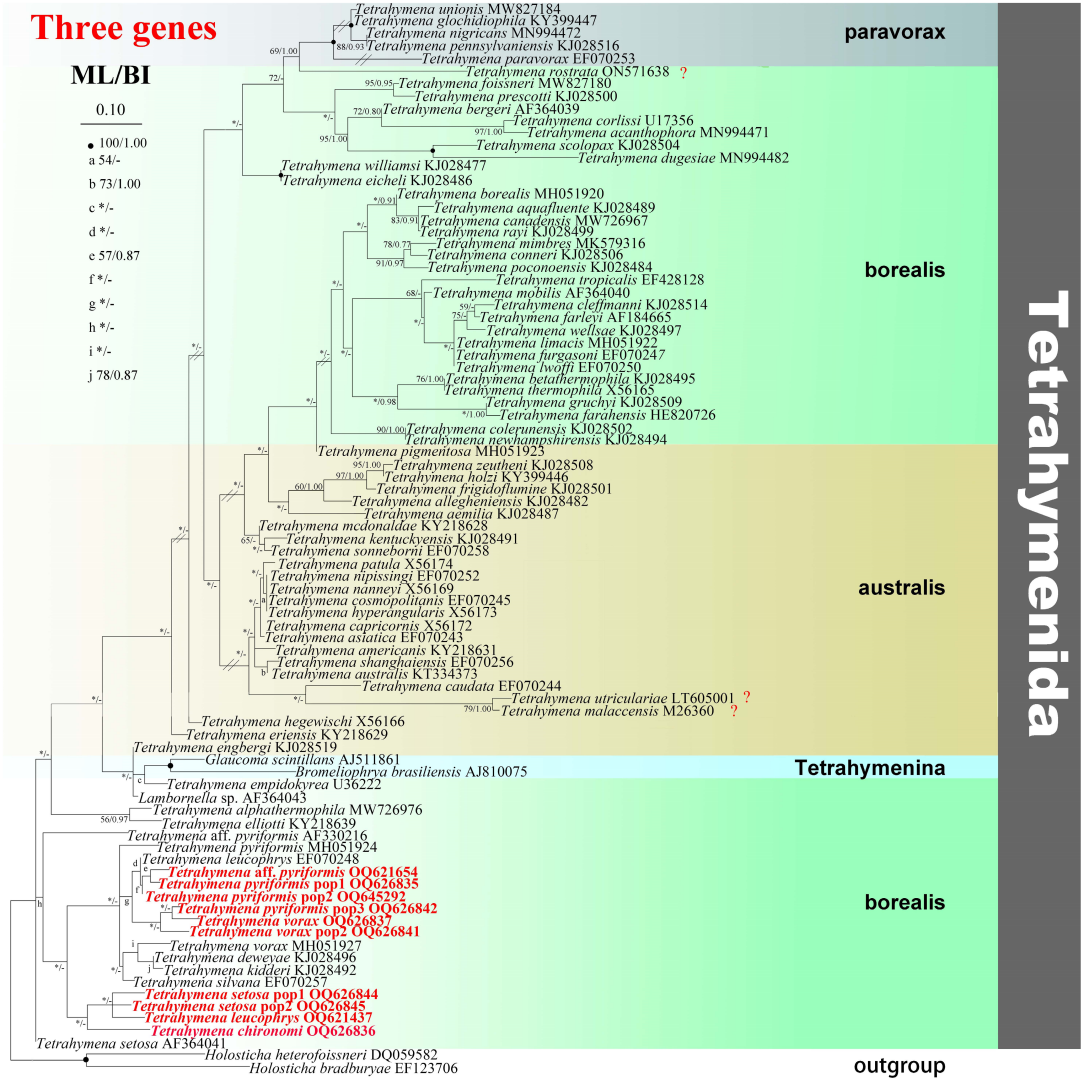
The maximum‐likelihood (ML) tree based on the concatenated three genes (18S rRNA, 5.8S‐rRNA genes, 28S rRNA) of representative members of the order Tetrahymenida. Species newly sequenced in the present study are shown in red type. Numbers near nodes are non‐parametric bootstrap values for maximum‐likelihood out of 1000 replicates and posterior probability values for Bayesian inference (BI), respectively. Question marks indicate the species with no morphological data and their identifications cannot be checked. “‐” at nodes indicates disagreement between the two methods. “*” at nodes indicates the support values <50/0.5 (ML/BI). Fully supported (100%/1.00) branches are marked with solid circles. The scale bar corresponds to 0.1 expected substitutions per site.

**FIGURE 2:**
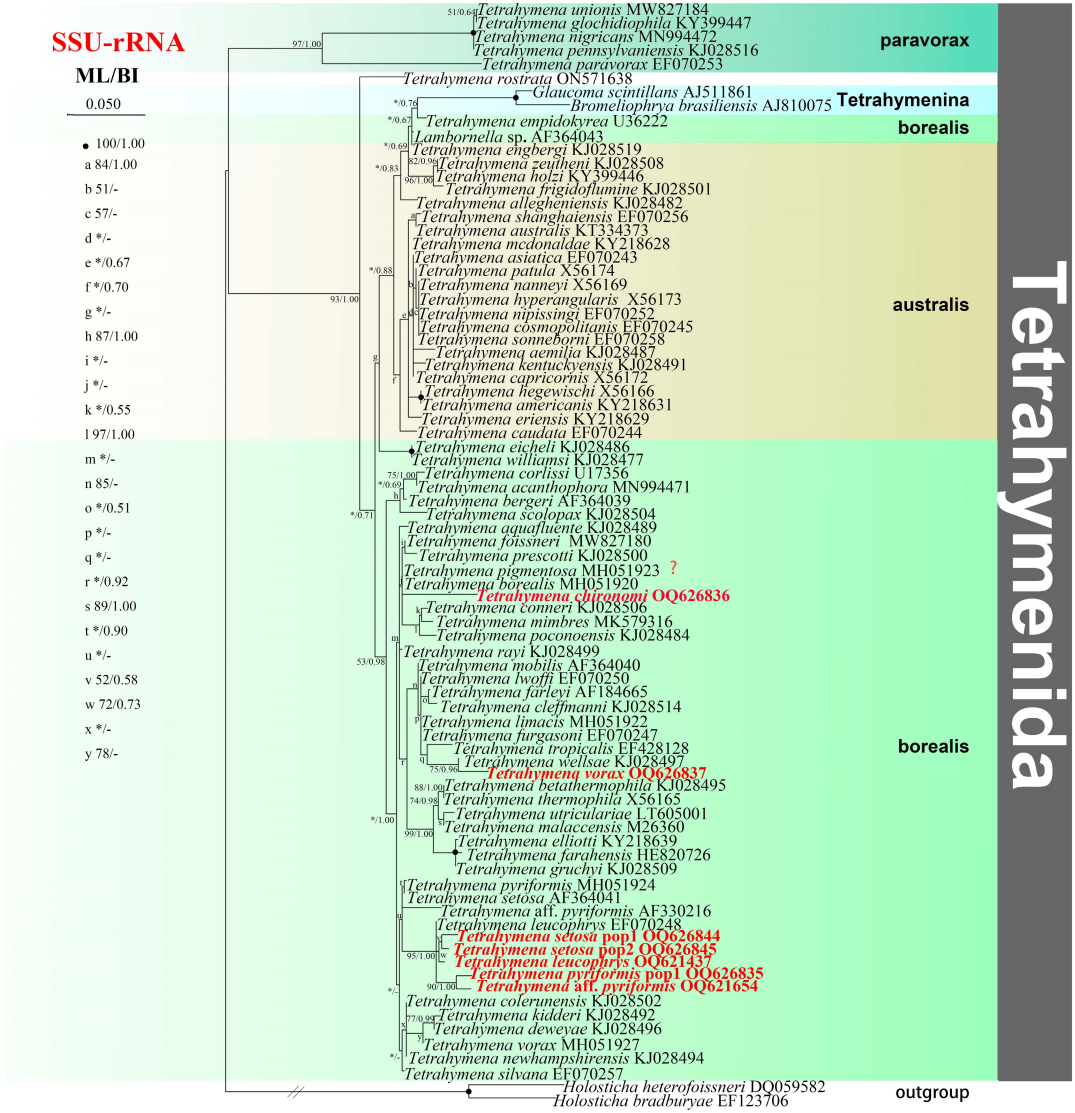
The maximum‐likelihood (ML) tree based on the 18S rRNA gene of representative members of the order Tetrahymenida. Species newly sequenced in the present study are shown in red type. Numbers near nodes are non‐parametric bootstrap values for maximum‐likelihood out of 1000 replicates and posterior probability values for Bayesian inference (BI), respectively. “‐” at nodes indicates disagreement between the two methods. Question marks indicate the species with no morphological data and their identifications cannot be checked. “*” at nodes indicates the support values <50/0.5 (ML/BI). Fully supported (100%/1.00) branches are marked with solid circles. The scale bar corresponds to 0.05 expected substitutions per site.

**FIGURE 3:**
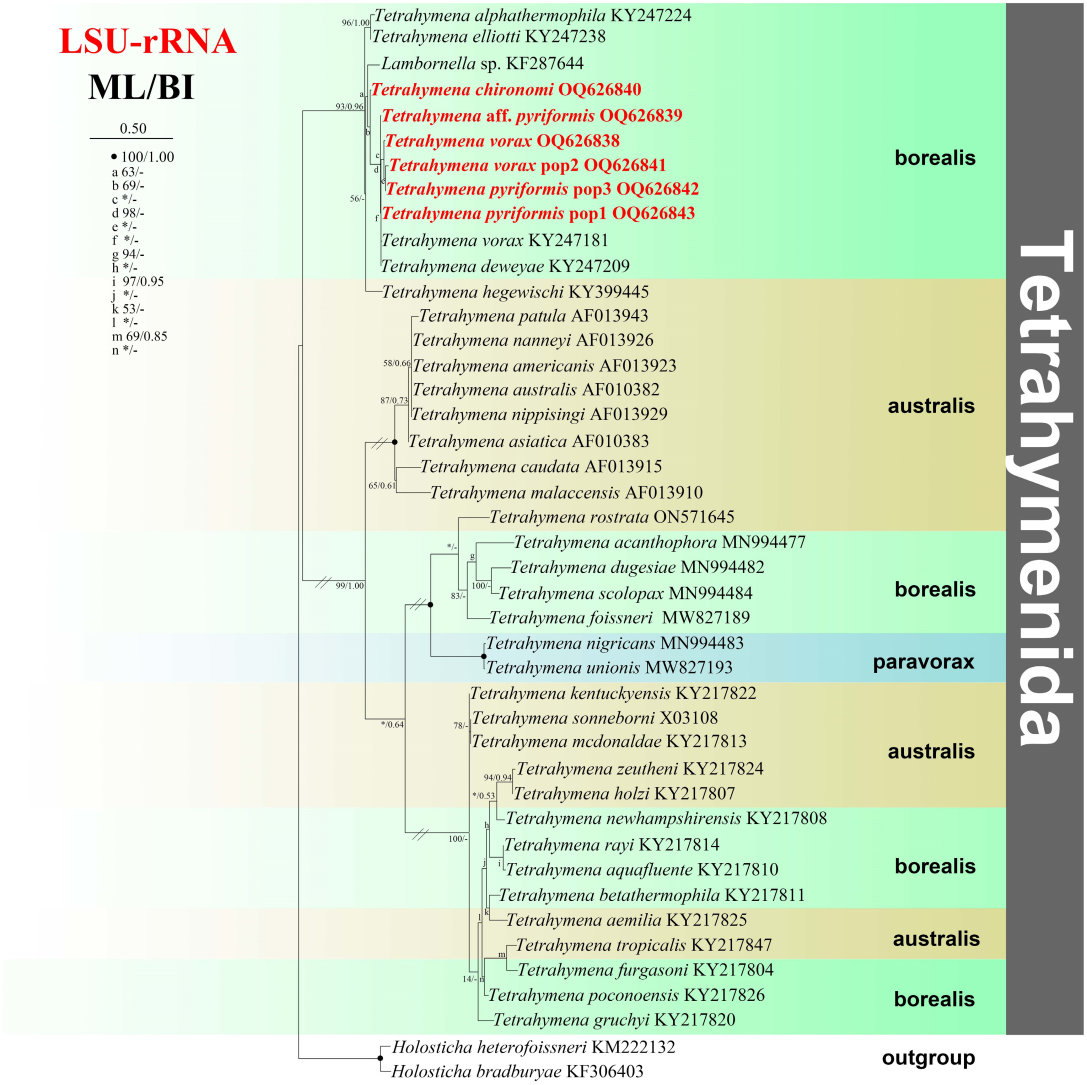
The maximum‐likelihood (ML) tree based on the 28S rRNA gene of representative members of the order Tetrahymenida. Species newly sequenced in the present study are shown in red type. Numbers near nodes are non‐parametric bootstrap values for maximum‐likelihood out of 1000 replicates and posterior probability values for Bayesian inference (BI), respectively. “‐” at nodes indicates disagreement between the two methods. “*” at nodes indicates the support values <50/0.5 (ML/BI). Fully supported (100%/1.00) branches are marked with solid circles. The scale bar corresponds to 0.5 expected substitutions per site.

**FIGURE 4:**
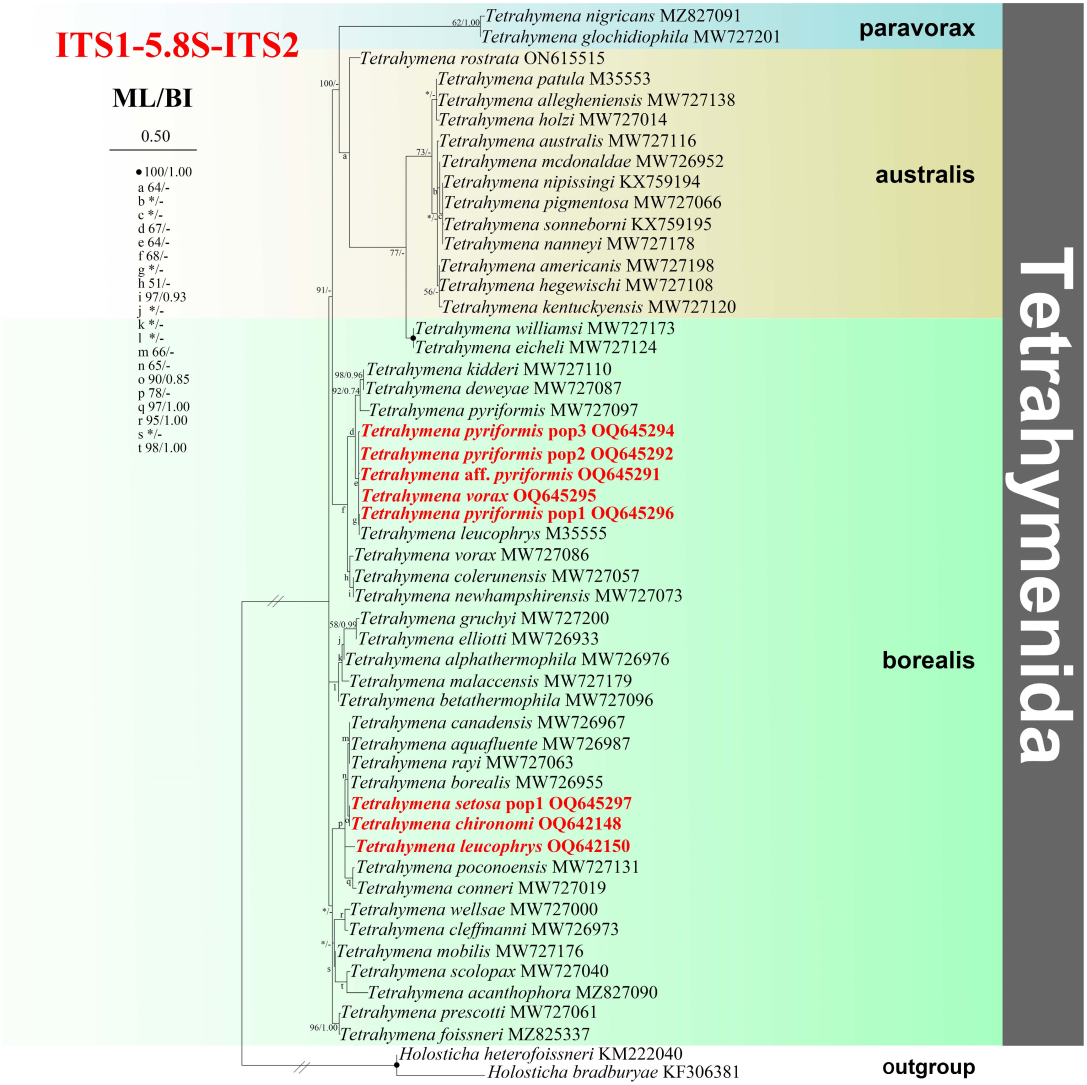
The maximum‐likelihood (ML) tree based on the ITS1‐5.8S‐ITS2 gene of representative members of the order Tetrahymenida. Species newly sequenced in the present study are shown in red type. Numbers near nodes are non‐parametric bootstrap values for maximum‐likelihood out of 1000 replicates and posterior probability values for Bayesian inference (BI), respectively. “‐” at nodes indicates disagreement between the two methods. “*” at nodes indicates the support values <50/0.5 (ML/BI). Fully supported (100%/1.00) branches are marked with solid circles. The scale bar corresponds to 0.1 expected substitutions per site.

In addition to the new sequences (Table 2), other sequences downloaded from GenBank database were used for phylogenetic analyses (shown in table, as shown in Figures 1, 2, 3, 4). All sequences are aligned using Clustal W in BioEdit 7.0.1 (Hall, 1999). The sequences for phylogenetic analysis were compiled into four data sets: (i) 1753 characters (81 taxa) of SSU‐rRNA gene; (ii) 1852 characters (43 taxa) of LSU‐rRNA gene; (iii) 1393 characters (52 taxa) of ITS1‐5.8S‐rRNA‐ITS2; (iv) 4237 characters (87 taxa in total) of the above three genes. In SeaView V4 (Manolo et al., 2012), these three alignments were linked to adjacent sites for gene analysis. All new sequences were stored in the NCBI database (see Table 2 for accession, length, and G&C content). The outgroup of all trees consists of three species of Holosticha (Holosticha Heterofoissneri, Holosticha Hradburyae).

**TABLE 2:** Newly sequenced genes in the present work.

Taxa	Accession numbers	Gene	Lengths (bp)	G&C%
*Tetrahymena vorax*	OQ626837	SSU rRNA	1688	42.95%
*Tetrahymena vorax*	OQ626838	LSU rRNA	1408	44.88%
*Tetrahymena vorax*	OQ645295	ITS1‐5.8S‐ITS2 rRNA	537	40.03%
*Tetrahymena vorax* pop2	OQ626841	LSU rRNA	1412	45.04%
*Tetrahymena leucophrys*	OQ621437	SSU rRNA	1691	43.11%
*Tetrahymena leucophrys*	OQ642150	ITS1‐5.8S‐ITS2 rRNA	541	40.48%
*Tetrahymena chironomi*	OQ626836	SSU rRNA	1689	43.10%
*Tetrahymena chironomi*	OQ626840	LSU rRNA	1425	44.42%
*Tetrahymena chironomi*	OQ642148	ITS1‐5.8S‐ITS2 rRNA	542	339.48%
*Tetrahymena* aff. *pyriformis*	OQ621654	SSU rRNA	1694	43.38%
*Tetrahymena* aff. *pyriformis*	OQ626839	LSU rRNA	1414	45.04%
*Tetrahymena* aff. *pyriformis*	OQ645291	ITS1‐5.8S‐ITS2 rRNA	540	40.37%
*Tetrahymena pyriformis* pop1	OQ626835	SSU rRNA	1694	43.03%
*Tetrahymena pyriformis* pop1	OQ626843	LSU rRNA	1412	44.90%
*Tetrahymena pyriformis* pop1	OQ645296	ITS1‐5.8S‐ITS2 rRNA	539	40.07%
*Tetrahymena pyriformis* pop2	OQ645292	ITS1‐5.8S‐ITS2 rRNA	540	40.18%
*Tetrahymena pyriformis* pop3	OQ626842	LSU rRNA	1405	45.12%
*Tetrahymena pyriformis* pop3	OQ645294	ITS1‐5.8S‐ITS2 rRNA	540	40.37%
*Tetrahymena setosa* pop1	OQ626844	SSU rRNA	1685	43.32%
*Tetrahymena setosa* pop1	OQ645297	ITS1‐5.8S‐ITS2 rRNA	541	39.55%
*Tetrahymena setosa* pop2	OQ626845	SSU rRNA	1692	43.08%

Phylogenetic trees were inferred and analyzed using maximum likelihood (ML) and Bayesian inference (BI). ML analysis was constructed by RAxML‐HPC2 v8.2.12 (Stamatakis, 2014), BI analysis was constructed by MrBayes v3.2.7a (Ronquist et al., 2012). All at CIPRES Science Gateway (URL: http://www.phylo.org/sub_sections/portal). The most suitable model for phylogenetic analysis of the nSSU‐rRNA gene (SSU‐rRNA, ITS1‐5.8S‐ITS2 rRNA, LSU‐rRNA genes) was GTR + I + G selected by Modeltest v3.4 (Posada & Crandall, 1998). According to the GTR + I + G model selected by MrModeltest v.2.2 program (Nylander, 2004). Bayesian posterior probabilities were computed by running four chains for 10,000,000 generations, with the cold chain sampling every 10,000 generations and the first 25% of sampled trees were discarded as “burn‐in.”

## 3 RESULTS

### 3.1 Phylogenetic analyses based on concatenated gene sequence data

The phylogenetic trees using two different methods (ML and BI) share basically congruent topologies. Therefore, only the ML trees are shown with support values from both methods (Figure 1). According to the concatenated gene phylogeny, Tetrahymenida clade comprises three major groups: borealis group, *paravorax* group, and australis group. The borealis group comprises two major sub‐groups. In borealis sub‐group I, the clade formed by *T. foissneri* MW827180, *T. prescotti* KJ028500, *T. bergeri* AF364039, *T. corlissi* U17356, *T. acanthophora* MN994471, and *T. scolopax* KJ028504 clusters with species of “*paravorax*” group, while the clade containing species for example, *T. borealis*, *T. lwoffi* EF070250, *T. furgasoni* EF070247, *T. limacis* MH051922, *T. farleyi* AF184665, *T. cleffmanni* KJ028514 and *T. wellsae* KJ028497 clusters with species of “australis” group. More specifically, *T. tropicalis* EF428128, *T. mobilis* AF364040. *T. aquafluente* KJ028489, *T. canadensis* MW726967, *T. rayi* KJ028499, and *T. borealis* MH051920 group together, the clade of which then forms a sister clade relationship with the clade grouped by *T. mimbres* MK579316, *T. conneri* KJ028506, *T. poconoensis* KJ028484. The borealis sub‐group II groups in the basal place of Tetrahymenida, and all the newly sequenced species group in this sub‐group. Noticeably, *T. pyriformis* pop3 forms a sister relationship with *T. vorax*, the clade of which then forms a sister relationship with *T. vorax* pop2. The sister clades (*T. pyriformis* pop1 + *T*. aff. *pyriformis* + *T. pyriformis* pop2) form a parallel clade with *T. leucophrys* EF070248. Four species *T. leucophrys*, *T. setosa* pop2, *T. setosa* pop1, and *T. chironomid* lies in the periphery of the clade.

The australis group cluster with borealis sub‐group I. In this group, *Tetrahymena zeutheni* KJ028508 clusters with *T. holzi* KY399446 with high support (95% ML, 1.00 BI), the clade of which then forms a sister relationship with *T. frigidoflumine* KJ028501. *T. asiatica* EF070243 forms a sister relationship with *T. capricornis* X56172, the clade of which then forms a sister relationship with the clade containing *T. hyperangularis* X56173 and *T. cosmopolitanis* EF070245.

Noticeably, all the parasitic and facultative parasitic species are clustered in borealis group.

### 3.2 Phylogenetic analyses based on SSU‐rRNA gene sequence data

The phylogenetic trees using two different methods (ML and BI) share basically congruent topologies, therefore, only the ML tree is shown with support values from both algorithms on branches (Figure 2). As shown in the figure, species assigned to Tetrahymenidae are mainly distributed into four parts: two borealis sub‐groups, one *paravorax* group and one australis group, and sequences of the paravorax group are nested within the borealis group.

The borealis group are also mainly distributed into two parts: one clade contains *T. furgasoni* EF070247, *T. limacis* MH051922, *T. lwoffi* EF070250, *T. cleffmanni* KJ028514, and *T. farleyi* AF184665, the clade of which then clusters with the clade grouped by *T. tropicalis* EF428128, *T. wellsae* KJ028497, and *T. vorax*. *T. conneri* KJ028506, *T. mimbres* MK579316, and *T. poconoensis* KJ028484 group together, and the clade of which then forms a parallel clade relationship with the sister clades (*T. prescotti* KJ028500 + *T. foissneri* MW827180), and the clade of which then forms a parallel clade relationship with *T. pigmentosa* MH051923, *T. chironomi*, *T. borealis* MH051920. In the other clade: *T. pyriformis* pop1 forms a sister relationship with *T*. aff. *pyriformis*, the clade of which then forms a sister relationship with *T. setosa* pop1, *T. setosa* pop2, and *T. leucophrys*. The periphery is *T. leucophrys* EF070248, and then *T*. aff. *pyriformis* AF330216 and *T. setosa* AF364041 are parallel clade. the clade of which then clusters with *T. kidderi* KJ028492, *T. deweyae* KJ028496, *T. vorax* MH051927, *T. newhampshire* KJ02849, *T. colerunensis* KJ028502, and *T. silvana* EF070257. *T. elliotti* KY218639, *T. gruchyi* KJ028509, *T. farahensis* HE820726 group with full support. The clade of which then clustered with the clade formed by *T. betathermophila* KJ028495 and *T. thermophila* X56165.

The australis group clusters with the borealis group and Tetrahymenina. *T. engbergi* KJ028519, *T. zeutheni* KJ028508, *T. holzi* KY399446, *T. frigidoflumine* KJ028501, and *T. allegheniensis* KJ028482 group with Tetrahymenina clade. *Tetrahymena shanghaiensis* EF070256, *T. australis* KT334373, *T. hyperangularis* X56173, *T. cosmopolitanis* EF070245, *T. capricornis* X56172, *T. aemilia* KJ028487, *T. mcdonaldae* KY218628, and other 11 *Tetrahymena* species group together with strong support, and the periphery is *T. caudata* EF070244.

The *paravorax* group cluster as a separate clade, and is sister to the clade formed by species from australis group, borealis group, and Tetrahymenina. *T. glochidiophila* KY399447 forms a sister relationship with *T. unionis* MW827184, the clade of which then clusters with *T. nigricans* MN994472 and *T. pennsylvaniensis* KJ028516 with full support. *T. paravorax* EF070253 occupies the basal position of the *paravorax* group.

It is consistent with concatenated tree that both parasitic and facultative parasitic species cluster in borealis group.

### 3.3 Phylogenetic analyses based on LSU‐rRNA gene sequence data

The phylogenetic trees using two different methods (ML and BI) share basically congruent topologies. Therefore, only the ML tree is shown. The topology of the LSU rRNA gene tree (Figure 3) is different from those of the above three trees. According to the LSU rRNA gene phylogeny, species assigned to Tetrahymenidae are mainly distributed into eight parts: four borealis sub‐groups, one *paravorax* group and three australis sub‐groups. Noticeably, all the newly sequenced species (*Tetrahymena vorax* pop2, *T. vorax*, *T. pyriformis* pop3, *T*. aff. *pyriformis*, *T. pyriformis* pop1, T. *chironomi*) cluster in the borealis group.

The *paravorax* group: *T. nigricans* MN994483 clusters with *T. unionis* MW827193 with full support.

The australis group: including *T. aemilia* KY217825, *T. zeutheni* KY217824, *T. holzi* KY217807, and *T. mcdonaldae* KY217813.

All the parasitic and facultative parasitic species cluster in borealis group.

### 3.4 Phylogenetic analyses based on ITS1‐5.8S‐ITS2 Region sequence data

The phylogenetic trees using two different methods (ML and BI) share basically congruent topologies (Figure 4). Therefore, only the ML tree is shown with the supported values from algorithms on the clade. According to the ITS1‐5.8S‐ITS2 rRNA phylogeny, species assigned to Tetrahymenidae are mainly distributed into three parts: one borealis groups, one *paravorax* group, and one australis group.

The borealis group are mainly distributed into six parallel clades. The first parallel clade: *T. williamsi* MW727173 forms a sister relationship with *T. eicheli* MW727124 with full support, the clade of which then groups with the australis group. The second parallel clade: *T. vorax* MW727086, *T. colerunensis* MW727057, *T. newhampshirensis* MW727073 group together, and then *T. vorax*, *T. pyriformis* pop2, *T. pyriformis* pop3, *T*. aff. *pyriformis*, and *T. pyriformis* pop1 form a parallel clade, the clade of which then clusters with (*T. kidderi* MW727110 + *T. deweyae* MW727087) clade (98% ML, 0.96 BI), and *T. leucophrys* M35555. The third parallel clade comprise *T. betathermophila*, *T. alphathermophila*, *T. gruchyi*, and *T. elliotti*. The fourth clade: the clade grouped by *T. poconoensis* MW727131 and *T. conneri* MW727019 forms a parallel clade relationship with *T. borealis* MW726955, *T. canadensis* MW726967, *T. aquafluent* MW726987, *T. rayi* MW727063, *T. leucophrys*, *T. setosa* pop1, and *T. chironomid* with strong support. The fifth parallel clade: *T. cleffmanni* MW726973 forms a sister relationship with *T. wellsae* MW727000 (95% ML, 0.99 BI), and then group with *T. mobile* MW727176, then *T. acanthophora* MZ827090, *T. scolopax* MW727040 are grouped at the periphery in turn. The sixth parallel clade: *T. prescotti* MW727061 is sister to *T. foissneri* MZ825337 with strong support (96% ML, 1.00 BI).

The *paravorax* group: *T. nigricans* MZ827091 clusters with *T. glochidiophila* MW727201.

The australis group: *T. holzi* MW727014 and *T. allegheniensis* MW727138 cluster with the clade grouped by *T. pigmentosa* MW727066 and *T. mcdonaldae* MW726952.

Both parasitic and facultative parasitic species cluster in the borealis group.

#### 3.4.1 Accession numbers

The data presented in the study are deposited in the NCBI database repository, accession numbers: *Tetrahymena vorax* OQ626837, *Tetrahymena vorax* OQ626838, *Tetrahymena vorax* OQ645295, *Tetrahymena vorax* pop2 OQ626841, *Tetrahymena leucophrys* OQ621437, *Tetrahymena leucophrys* OQ642150, *Tetrahymena chironomi* OQ626836, *Tetrahymena chironomi* OQ626840, *Tetrahymena chironomi* OQ642148, *Tetrahymena* aff. *pyriformis* OQ621654, *Tetrahymena* aff. p*yriformis* OQ626839, *Tetrahymena* aff. *pyriformis* OQ645291, *Tetrahymena pyriformis* pop1 OQ626835, *Tetrahymena pyriformis* pop1 OQ626843, *Tetrahymena pyriformis* pop1 OQ645296, *Tetrahymena pyriformis* pop2 OQ645292, *Tetrahymena pyriformis* pop3 OQ626842, *Tetrahymena pyriformis* pop3 OQ645294, *Tetrahymena setosa* pop1 OQ626844, *Tetrahymena setosa* pop1 OQ645297, *Tetrahymena setosa* pop2 OQ626845.

## 4 DISCUSSION

### 4.1 Phylogeny of *Tetrahymena*

In the present study, 21 new multiple gene sequences of 10 populations from five *Tetrahymena* species are added, including three populations of *T. pyriformis*, two populations of *Tetrahymena vorax* and *T. setosa*, and one population of *T. leucophrys*, *T. chironomid*, and *T*. aff. *pyriformis*. The phylogenetic trees deduced from the three different methods (18S rRNA, ITS1‐5.8S‐ITS2, and concatenated gene) showed quite similar topologies. The divergence of topology of the LSU rRNA gene with those of other genes might due to the shortage of the gene sequencing. With the addition of new sequences, *Tetrahymena* species is still mainly classified into three groups, namely the “borealis group,” the “australis group,” and the “*paravorax* group.” Phylogeny based on concatenated genes shows a similar result with previous studies (Chantangsi et al., 2007; Liu et al., 2016; Pan et al., 2020). However, it is speculated that gene sequencing remains unbalanced in this group, especially for LSU rRNA genes.

With the addition of new sequences, *Tetrahymena* species is still mainly classified into three groups, namely the “borealis group,” the “australis group,” and the “*paravorax* group.” There are some similarities between the members of each group in terms of morphological characteristics or life cycle patterns: borealis group species usually has a smaller body and fewer somatic kinetics, bacterivorous; australis group species usually has a larger body, more somatic kinetics, and the ability to form resting cysts, some australis group members can undergo microstome–macrostome transformation (Corliss, 1973). While we agree that morphological characters are important, the results of Lynn et al. (2018) once again demonstrate that morphology fails to distinguish among *Tetrahymena* species.

Additionally, *T. utriculariae* LT605001 and *T. malaccensis* M26360 group within the australis group, although they were morphologically assigned in the borealis group. Since the researcher did not provide any morphological information about the population of *T. utriculariae* LT605001 and *T. malaccensis* M26360, thus the identifications of this two sequences cannot be checked.

### 4.2 Phylogeny of parasitic or facultative parasitic *Tetrahymena*

*Tetrahymena vorax* and *T. pyriformis* are usually regarded as free‐living forms, whereas in the present work they were isolated from skin, surface mucus, gills, and internal organs of diseased fish for the first time. A possible reason for this is that in high latitude cold areas these species may have multiple life‐styles (facultative parasitic and free‐living) due to the slow degradations of microorganisms and insufficiency of food (Pan et al., 2020, 2022). *Tetrahymena* species are classified as three groups, namely the “borealis group,” the “australis group,” and the “*paravorax* group” (Chantangsi et al., 2007; Liu et al., 2016; Pan et al., 2020). The clustering patterns of all the newly sequenced parasitic or facultative parasitic *Tetrahymena* species (Figures 1, 2, 3, 4) show that members of the “borealis” group may be more susceptible to parasitism (Chantangsi et al., 2007; Pan et al., 2020). However, a deeper elucidation of phylogenetic relationships among Tetrahymenida will certainly need much wider taxon coverage.

### 4.3 Systematic evolution position of *Tetrahymena vorax*

Both LSU rRNA and ITS1‐5.8S‐ITS2 rRNA gene trees show that *T. vorax* groups with Harbin populations of *T. vorax* and *T. pyriformis*, while *T. vorax* clusters with *T. wellsae* in the SSU rRNA gene tree. However, phylogeny based on concatenated genes shows that *T. vorax* MH051927, *T. kidderi* KJ028492, and *T. deweyae* KJ028496 group together.

The reasons for the different systematic positions of Harbin population of *T. vorax* with other populations of *T. vorax* are: (1) There are no morphological information for other populations of *T. vorax*, and the species might not be correctly identified, (2) the molecular data available in the NCBI database for *T. vorax* is very sparse, and broader sampling and molecular sequencing of this species is needed (Chantangsi, 2006; Chantangsi et al., 2007).

### 4.4 Systematic evolution position of *Tetrahymena chironomi*

Phylogeny based on the SSU rRNA gene shows that *T. chironomi* clusters with *T. pigmentosa* MH051923 and *T. borealis* MH051920, while 5.8S rRNA gene and concatenated gene trees show that *T. chironomi* clusters with *T. setosa*. The facts above show the uncertain location of *T. chironomi* (Sun, 2021). This may be due to the limited genetic information contained by a single gene, it is not possible to clearly distinguish the species with close genetic relationship, while combining several factors can provide more sufficient genetic information.

### 4.5 Systematic evolution position of *T. pyriformis*, *T. setosa*, and *T. leucophrys*

In the LSU rRNA gene, 5.8S gene and concatenated gene trees, *T. pyriformis* always clusters with *T. vorax*. However, in the SSU rRNA tree, *T. pyriformis* clusters with *T. setosa*. In our results, phylogeny based on SSU rRNA gene shows that *T. setosa* AF364041 and *T*. aff. *pyriformis* AF330216 form parallel clades. In the concatenated genes phylogenetic tree, *T. setosa* grouped at the periphery. Pan et al. (2018) analyzed the relationship within *Tetrahymena* species based on the SSU rRNA gene, in their work, *T. pyriformis* always grouped with *T. setosa* and *T. leucophrys*. The results of phylogeny based on concatenated genes and SSU rRNA gene are consistent with those of the previous studies (Chantangsi et al., 2007; Chantangsi & Lynn, 2008; Kypke & Lynn, 2010; Liu et al., 2016; Pan et al., 2018; Rataj & Vdacny, 2020). Based on the above results, we agree with the results of the concatenated genes phylogenetic tree that *T. pyriformis*, *T. setosa*, and *T. leucophrys* have close relationship.

### 4.6 Systematic evolution position of *T. rostrata*

In the present work, *Tetrahymena rostrata* clusters with species of the borealis group, and separate with other “australis” group species in the concatenated genes tree, the result of which is not consistent with present studies (Chantangsi et al., 2007; Liu et al., 2016; Lynn et al., 2018; Pan et al., 2018). According to Corliss (1973), *T. rostrata* is a parasitic and histophagous species. Based on the present result of concatenated genes tree and its parasitic habit, we propose that *T. rostrata* should be assigned to the borealis group, but not the australis group.

### 4.7 Systematic evolution position of *Tetrahymena pigmentosa*

*Tetrahymena pigmentosa* clusters in the australis group, which is consistent with previous studies (Chantangsi et al., 2007; Liu et al., 2016; Lynn et al., 2018; Pan et al., 2018; Rataj & Vdacny, 2020; Sun et al., 2020). The results of positions of *T. pigmentosa* based on concatenated genes (SSU rRNA, ITS1‐5.8S‐ITS2, and LSU rRNA), LSU rDNA gene and 5.8S gene are also consistent. However, in SSU rRNA gene tree, *T. pigmentosa* MH051923 clusters in borealis group, the phylogenic result is inconsistent with those of other genes. Based on the result of multiple genes in the present result, we agree with the previous reports that *T. pigmentosa* should belong to australis group. *T. pigmentosa* MH051923 is probably in fact *T. chironomi*, since the researcher did not provide any morphological information about the population of *T. pigmentosa* MH051923 thus the identification cannot be checked. Obviously, many new taxa are still awaiting discovery in order to elucidate the systematics of the genus *Tetrahymena*, although many forms have been found.

## AUTHOR CONTRIBUTIONS

**Lihui Liu:** Formal analysis (lead); software (lead); writing – original draft (lead); writing – review and editing (equal). **Mingyue Jiang:** Formal analysis (equal); investigation (equal); software (equal). **Chunyu Zhou:** Formal analysis (equal); investigation (equal); software (equal). **Bailin Li:** Formal analysis (equal); investigation (equal); software (equal). **Yumeng Song:** Formal analysis (equal); investigation (equal); software (equal). **Xuming Pan:** Conceptualization (lead); formal analysis (lead); funding acquisition (lead); methodology (lead); supervision (lead); writing – review and editing (equal).

## CONFLICT OF INTEREST STATEMENT

All authors declare that they have no conflicts of interest.

## Data Availability

The data presented in the study are deposited in the NCBI database (https://www.ncbi.nlm.nih.gov/) repository, accession numbers, lengths, and G&C contents are shown in Table 2. It maybe not traceable until the date release.
